# Utilization of Women’s Preventive Health Services During the COVID-19 Pandemic

**DOI:** 10.1001/jamahealthforum.2021.1408

**Published:** 2021-07-02

**Authors:** Nora V. Becker, Michelle H. Moniz, Renuka Tipirneni, Vanessa K. Dalton, John Z. Ayanian

**Affiliations:** 1 Division of General Medicine, University of Michigan Medical School, Ann Arbor; 2 Institute for Healthcare Policy and Innovation, University of Michigan, Ann Arbor; 3 Program on Women’s Healthcare Effectiveness Research, University of Michigan Medical School, Ann Arbor; 4 Department of Obstetrics and Gynecology, University of Michigan Medical School, Ann Arbor; 5 Editor, *JAMA Health Forum*

## Abstract

**Importance:**

The association of the COVID-19 pandemic with women’s preventive health care use is unknown.

**Objective:**

To describe utilization of women’s preventive health services.

**Design, Setting, and Participants:**

Cross-sectional study of women aged 18 to 74 years enrolled in a commercial health maintenance organization in Michigan.

**Exposures:**

COVID-19 pandemic (2019-2020).

**Main Outcomes and Measures:**

Adjusted odds ratios (AORs) of receiving breast cancer screening, cervical cancer screening, sexually transmitted infection (STI) screening, long-acting reversible contraception (LARC) insertions, and pharmacy-obtained contraception, adjusted for month, age, county, zip code characteristics (per-capita income, non-White percentage of population, non–English-proficient percentage of population), and plan designation (primary plan holder vs dependent).

**Results:**

The study population included 685 373 women aged 18 to 74 years, enrolled for 13 000 715 person-months, of whom 10 061 275 person-months (77.4%) were among women aged 25 to 64 years and 8 020 215 (61.7%) were the primary plan holder, with mean zip code per capita income of $33 708, 20.2% mean zip code non-White population, and 3.4% mean zip code non–English-speaking population. For services requiring an in-person visit (breast cancer screening, cervical cancer screening, STI testing, and LARC insertions), utilization declined by 60% to 90% during the spring of 2020, with a nadir in April 2020, after which utilization for all services recovered to close to 2019 levels by July 2020. Claims for pharmacy-obtained hormonal contraceptives in 2020 were consistently 15% to 30% lower than 2019. The AORs of a woman receiving a given preventive service in 2020 compared with 2019 were significantly lower for breast cancer screening (AOR, 0.80; 95% CI, 0.79-0.80), cervical cancer screening (AOR, 0.80; 95% CI, 0.80-0.81), STI screening (AOR, 0.83; 95% CI, 0.82-0.84), LARC insertion (AOR, 0.87; 95% CI, 0.84-0.90), and pharmacy-obtained contraception (AOR, 0.73; 95% CI, 0.72-0.74) (all *P* < .001).

**Conclusions and Relevance:**

In this cross-sectional study of women enrolled in a large US commercial health maintenance organization plan, the COVID-19 pandemic was associated with large but transient declines in rates of breast cancer screening, cervical cancer screening, STI screening, and LARC insertions, and moderate persistent declines in pharmacy-obtained hormonal contraceptives. The overall odds of a woman receiving a given preventive service in 2020 was 20% to 30% lower than 2019. Further research into disparities in access to care and the health outcomes of decreased use of these key health services is warranted.

## Key Points

**Question:**

How has utilization of preventive health services among commercially insured women changed during the COVID-19 pandemic?

**Findings:**

In this cross-sectional study of 685 373 women enrolled in a large commercial health maintenance organization plan in Michigan, rates of breast cancer screening, cervical cancer screening, sexually transmitted infection screening, and prescriptions and new insertions for contraceptives all significantly declined in 2020 compared with 2019. For some services, there were small declines in utilization among women residing in zip codes that are lower income with higher proportions of non-White and non–English-speaking residents.

**Meaning:**

The pandemic has disrupted the utilization of women’s preventive health services and may be associated with increased disparities in access to these services.

## Teaser

This cross-sectional study describes the utilization of women’s preventive health services during the COVID-19 pandemic.

## Introduction

The use of recommended preventive health services is an important driver of women’s health outcomes and is associated with decreased rates of breast and cervical cancer mortality, lower rates of unwanted pregnancy and abortion, and appropriate treatment of sexually transmitted infections (STIs).^1^ Women’s utilization of preventive health services was made more accessible and affordable for women by the implementation of the Affordable Care Act (ACA), which expanded insurance coverage, prevented gender-based premium pricing, and mandated that commercial health plans cover effective preventive services with no out-of-pocket costs to patients. These policies have been associated with improvement in women’s utilization of preventive health services in recent years.^2,3^

The SARS-CoV-2 (COVID-19) pandemic has been associated with numerous disruptions in routine medical care,^4^ but little is known about the influence of these disruptions on preventive health care for women. One study using national commercial insurance claims found that mammography rates declined by 67% in March and April of 2020 compared with the same months in 2019.^5^ However, utilization of other preventive health services for women during the pandemic has not been studied, and utilization in the latter half of 2020 has not been described. If the pandemic has caused significant disruption of women’s health care, recent gains in access to and utilization of women’s preventive services under the ACA could be blunted or reversed.

Michigan experienced a large early wave of COVID-19 cases in March through May 2020. By June, COVID-19 case rates in the state had fallen dramatically and remained low over the summer until early fall, when a second wave of infections began.^6^ The spring surge of COVID-19 cases was accompanied by widespread suspension of nonemergency medical care across the state.^7^ The statewide response to the fall surge did not precipitate similar care disruptions. Our objective was to evaluate potential changes in women’s preventive service use during the pandemic in a large commercially insured cohort.

## Methods

### Data Source and Study Population

We used medical and pharmacy claims data from enrollees in the commercial health maintenance organization (HMO) plan of Blue Cross Blue Shield of Michigan (BCBSM), a large insurance plan with approximately 1 million monthly enrollees statewide. Claims for all women enrollees aged 18 to 74 years from January 2019 to January 2021 were used to identify women’s preventive health services. Individuals older than 65 years were included if they remained employed, had their primary insurance through an employed spouse, or had purchased a supplementary Medicare plan (Medigap) within the BCBSM HMO network. Medicare Advantage enrollees and traditional Medicare enrollees without Medigap coverage through the BCBSM HMO network were not included in the data. The data were accessed via the Michigan Value Collaborative, a partnership between Michigan hospitals and BCBSM. Support for the Michigan Value Collaborative is provided by BCBSM as part of the BCBSM Value Partnerships program; however, the opinions, beliefs, and viewpoints expressed by the authors do not necessarily reflect those of BCBSM or any of its employees. Importantly, BCBSM and its employees were not involved in any aspect of the study at any point. This project was deemed exempt from review and informed consent by the University of Michigan Institutional Review board owing to the use of deidentified data.

The data source includes comprehensive medical and pharmaceutical claims for all enrollees in the health plan, as well as each enrollee’s gender, date of birth, zip code of residence, and whether they are the primary plan holder or a dependent (spouse, child, or other dependent). The data used for the study did not include information on race/ethnicity. The data are structured by the month of billing, not the month of service, so we reconstructed all utilization by service date prior to performing our analysis. Approximately two-thirds of the services delivered in a given month are billed in that same month, with another 30% billed in the subsequent month and about 5% of services billed 2 months or longer from the date of service. The final month of currently available data was January 2021, and therefore services that were delivered in December 2020 and billed in January 2021 are included in our data.

### Outcome Measures

Utilization of women’s preventive health services was identified using *International Classification of Diseases and Related Health Problems, Tenth Revision*, Current Procedural Terminology, and Healthcare Common Procedure Coding System codes within medical claims, and National Drug Codes within pharmacy claims (eAppendix 1 in the Supplement). Women’s preventive health services were classified as breast cancer screening, cervical cancer screening, screening for STIs, insertions of long-acting reversible contraceptives (LARCs), and pharmacy-obtained hormonal contraception.

Breast cancer screening was identified using diagnostic codes for screening mammography, and codes identifying diagnostic mammography were excluded. Cervical cancer screening was defined as any Papanicolaou test or human papillomavirus test. Screening for STIs was identified using general diagnostic codes for screening and counseling for high-risk sexual behavior as well as specific codes for chlamydia, gonorrhea, HIV, and syphilis testing. Insertions of LARC were identified using codes for intrauterine device and subdermal contraceptive implant insertions. Pharmacy claims were used to identify filled prescriptions for oral contraceptive pills, transdermal contraceptive patches, and contraceptive vaginal rings.

To calculate a relevant monthly claims rate for each service, we defined the typical age range of utilization for each medical service, using generally accepted screening guidelines when available. The age ranges for each service were 40 to 74 years for mammograms,^8,9^ 21 to 65 years for cervical cancer screening,^10^ 18 to 74 years for STI testing,^11,12,13^ and 18 to 45 years for LARC insertions and pharmacy-obtained contraception. Monthly claims rates for each service were calculated as the number of claims for a medical service among female enrollees in that age range in that month, divided by the total number of enrolled women in that age range in that month. These monthly claims rates were examined visually to assess dynamic changes in utilization within each year.

### Other Covariates

Other individual-level covariates were not available in our data; however, there were potentially changes in the composition of the enrolled population during the period of the study, and we wished to control for these changes when estimating the change in utilization between 2019 and 2020. We therefore merged in 3 zip code–level estimates from the 2019 American Community Survey: per-capita income, percentage of population reporting non-White race, and percentage of population not proficient in English.^14^ We also assigned enrollees to counties in Michigan according to their zip code using a crosswalk available from the US Department of Housing and Urban Development. When zip codes crossed county boundaries, we assigned enrollees to the county where the majority of the residents within the zip code reside.^15^ Covariates were chosen a priori based on sociodemographic variables that have been shown to be correlated with use of cancer screening services.^16^

### Statistical Analysis

We calculated descriptive statistics for women in the study cohort. To compare overall rates of utilization from 2019 to 2020, we used logistic regression to estimate the adjusted odds ratios of utilization of each service in 2020 relative to 2019. Additional regression covariates include calendar month fixed effects, county fixed effects, plan holder status fixed effects (primary plan holder, spouse, child, or other dependent), age band fixed effects, zip code per-capita income, zip code percentage non-White population, and zip code percentage of the population not English proficient. Plan holder status was included as a covariate because it is associated with marital status and because many young women younger than 26 years are insured on their parent’s insurance, which may alter their choices regarding reproductive health service use. Robust standard errors were clustered at the individual level. Regressions exclude observations with missing covariates. As a secondary analysis, we also estimated adjusted odds ratios of the utilization of each service in the latter 6 months (July to December) of 2020 vs 2019, using the same covariates as previously stated.

To compare differences in the populations of women receiving preventive services during the pandemic, we performed a descriptive post hoc analysis comparing the distribution of covariates among women receiving each preventive service in the prepandemic (January 2019 to February 2020) vs postpandemic period (March to December 2020). For each covariate (age, zip code per-capita income, zip code percentage of non-White population, and zip code percentage of non–English-proficient population), we reported the mean and SD for women receiving each service in the prepandemic and postpandemic period, along with the *P* value of a 2-sided *t* test for statistical difference between the 2 means. All *P* values were adjusted for multiple comparisons using the Bonferroni correction; by convention, *P* values greater than 1 after correction were reported as *P* > .99.

All analyses were performed using Stata-MP statistical software, version 16 (StataCorp LLC). The study was reported according to the Strengthening the Reporting of Observational Studies in Epidemiology (STROBE) reporting guideline.^17^

## Results

**Figure 1.**
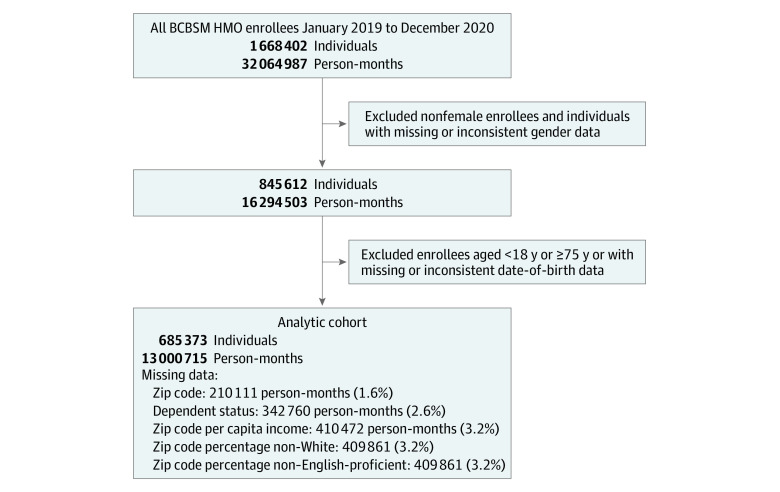
Analytic Cohort Construction Details of construction of analytic cohort and missing data. BCBSM indicates Blue Cross Blue Shield of Michigan; HMO, health maintenance organization.

The analytic cohort includes 685 373 women aged 18 to 74 years with 13 000 715 month-level observations during 2019 and 2020 (Table 1); 10 061 275 person-months (77.4%) were among women aged 25 to 64 years, and 8 020 215 person-months (61.7%) were among women who were the primary plan holder. The mean zip code per-capita income was $33 708, the mean zip code population percentage non-White was 20.2%, and the mean zip code population percentage not English proficient was 3.4%. Figure 1 describes the details of the analytic cohort construction, including rates of missing data for all covariates.

**Table 1.** Descriptive Statistics

Characteristic	No. (%) (n = 685 373)
Total person-months	13 000 715 (100)
Age group (person-months)	
18-24 y	1 557 188 (12.0)
25-34 y	2 766 853 (21.3)
35-44 y	2 146 491 (16.5)
45-54 y	2 371 866 (18.2)
55-64 y	2 776 065 (21.4)
65-74 y	1 382 252 (10.6)
Dependent status (person-months)	
Primary	8 020 215 (61.7)
Spouse	3 208 839 (24.7)
Child	1 424 805 (11.0)
Other dependent	4096 (0.03)
Mean zip code, mean (SE)	
Per-capita income, $	33 708.27 (10 775.52)
Non-White population, %^a^	20.2 (21.0)
Non–English-proficient population, %	3.4 (4.3)

**Figure 2.**
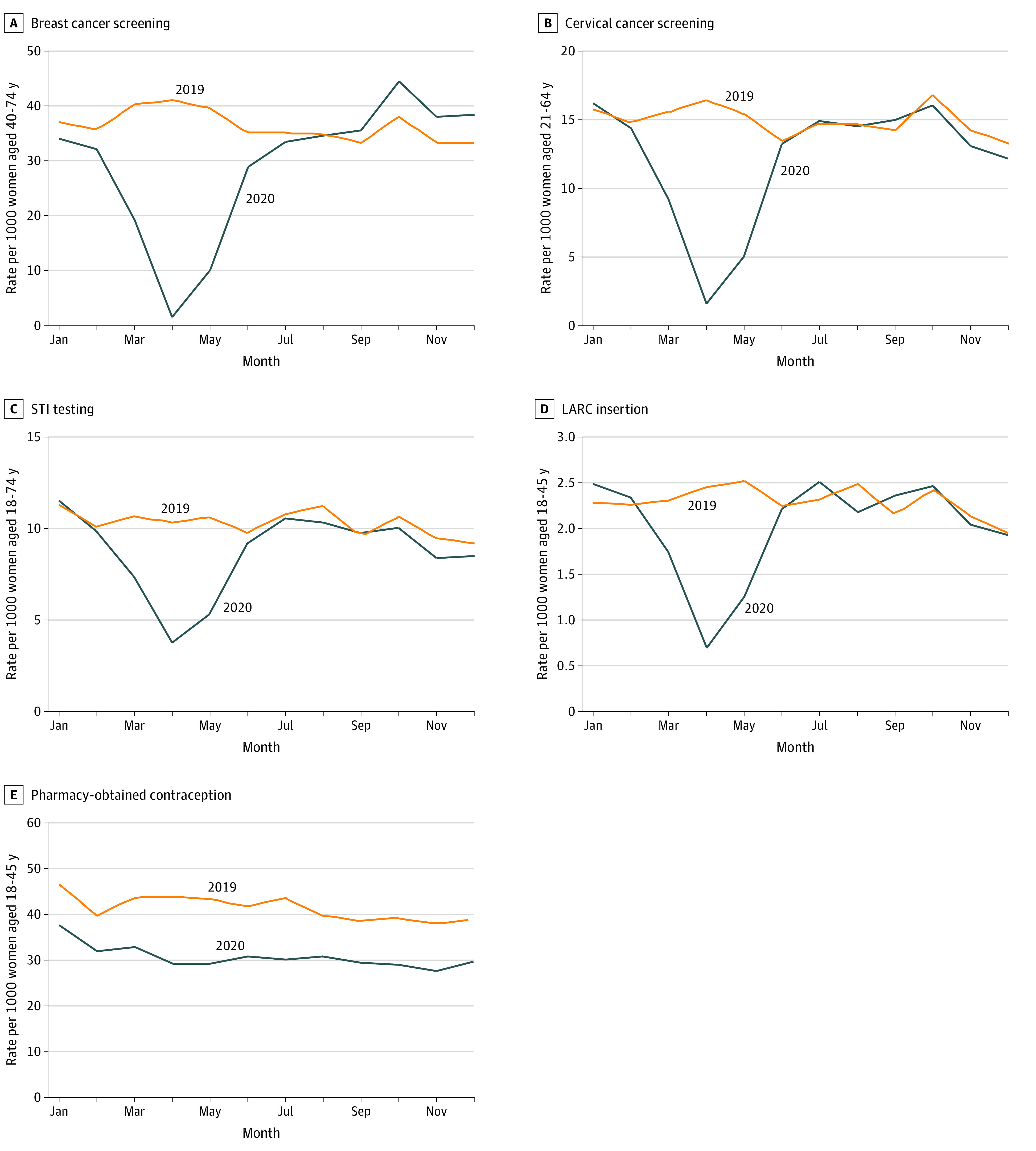
Women’s Preventive Services Claim Rates in 2019 and 2020 A and B, Monthly rates of breast and cervical cancer screening. C, D, and E, Monthly rates of sexually transmitted infection (STI) testing, long-acting reversible contraception (LARC) insertions, and pharmacy-obtained contraception.

Figure 2 displays monthly claim rates by service in 2019 and 2020. All services except pharmacy-obtained contraceptive demonstrated a sharp decline in utilization in the first half of 2020, with the lowest rates of use seen in April 2020, followed by a recovery by July 2020 compared with 2019 levels. The largest percentage declines in utilization were seen in breast and cervical cancer screening, which declined by 96.6% and 90.5%, respectively, in April 2020 relative to the prior year. A similar pattern was observed for STI testing and LARC insertion rates, which nadired at 63.5% and 71.6% declines in April 2020. For all of these services, rates of claims reverted to close to 2019 levels by July 2020. In contrast, claims for pharmacy-obtained contraceptives were consistently 15% to 30% lower throughout 2020 relative to 2019, without the significant temporal variation seen during the year with the other services.

Adjusted odds ratios of utilization of each type of service are shown in Table 2. The adjusted odds ratios for women in the specified age ranges receiving each of the preventive services was significantly lower in 2020 relative to 2019 (*P* < .001 for all estimates), with values ranging from an adjusted odds ratio of 0.73 (95% CI, 0.72-0.74) for pharmacy-obtained contraception to 0.87 (95% CI, 0.84-0.90) for LARC insertion.

**Table 2.** Adjusted Odds Ratios of Utilization of Women’s Preventive Health Services in 2020 Relative to 2019^a^

Preventive service	No.	Age range, y	Odds ratio in 2020 relative to 2019 (95% CI)	*P* value
Breast cancer screening	7 250 080	40-74	0.80 (0.79-0.80)	<.001
Cervical cancer screening	10 680 446	21-65	0.80 (0.80-0.81)	<.001
STI screening	12 325 776	18-74	0.83 (0.82-0.84)	<.001
LARC insertion	6 270 414	18-45	0.87 (0.84-0.90)	<.001
Pharmacy-obtained contraception	6 274 035	18-45	0.73 (0.72-0.74)	<.001

Abbreviations: LARC, long-acting reversible contraception; STI, sexually transmitted infection.

^a^ Each model predicts the adjusted odds ratio that an enrolled woman in the given age range has a claim for a given service in 2020 relative to 2019. Additional covariates include calendar month fixed effects, county fixed effects, dependent status fixed effects (primary plan holder, spouse, child, or other dependent), age group fixed effects, and three 2019 zip code–level covariates (per-capita income, percentage of population that is non-White, and percentage of population that is not English proficient). Robust standard errors clustered at the individual level.

For the 4 services that displayed a temporal drop and then recovery in their utilization rates, we also tested whether utilization in the second half of 2020 (July to December) was statistically significantly different compared with the same months in 2019. We found that only breast cancer screening recovered to a utilization level above the 2019 baseline (adjusted odds ratio, 1.08; *P* < .001), with all other services either slightly lower than 2019 utilization levels or not significantly different (eAppendix 2 in the Supplement).

Table 3 displays the mean values of covariates among women receiving each type of preventive service in the prepandemic (January 2019 to February 2020) vs postpandemic (March to December 2020) periods. There were some statistically significant but modest differences between women receiving each type of service in the prepandemic and postpandemic periods. Women receiving breast cancer screening in the postpandemic periods were slightly older (mean [SD] age, 55.8 [9.0] years in the prepandemic period vs 56.3 [9.0] years in the postpandemic period; *P* < .001) and resided in zip codes with slightly more English speakers (3.1% vs 3.0%, *P* < .001). Women receiving cervical cancer screening in the postpandemic period were slightly younger (mean [SD] age, 42.3 [13.4] years vs 42.1 [13.6] years; *P* = .03) and resided in zip codes with higher per-capita income ($34 777 vs $35 056; *P* < .001). Among women who received STI screening, women were slightly younger in the postpandemic period (mean [SD] age, 31.7 [11.6] years vs 31.4 [11.2] years; *P* = .002) and resided in zip codes with higher per-capita income ($32 981 vs $33 232; *P* = .004), lower percentages of non-White residents (25.7% vs 25%; *P* < .001), and lower percentages of non-English speakers (3.7% vs 3.6%; *P* = .02). There were no significant differences in any of the covariates among women receiving LARC insertions in the prepandemic vs postpandemic periods. Among women receiving contraception from a pharmacy, they were more likely to reside in zip codes with higher per-capita income ($34 625 vs $34 976; *P* < .001), and slightly higher percentages of non-White residents (18.4% vs 18.7%; *P* = .01).

**Table 3.** Distribution of Covariates Among Women Receiving Preventive Services Prepandemic vs Postpandemic

Preventive service^a^	No.	Age, y	Zip code per-capita income, $	Zip code percentage non-White, %	Zip code percentage non–English proficient, %
Breast cancer screening					
Prepandemic, mean (SD)	162 917	55.8 (9.0)	34 015 (10 706)	19.9 (21.4)	3.1 (4.0)
Postpandemic, mean (SD)	89 853	56.3 (9.0)	34 034 (10 726)	19.6 (21.5)	3.0 (3.8)
*P* value for *t* test	NA	<.001	>.99	.06	<.001
Cervical cancer screening					
Prepandemic, mean (SD)	102 484	42.3 (13.4)	34 777 (11 240)	20.4 (20.5)	3.5 (4.3)
Postpandemic, mean (SD)	54 906	42.1 (13.6)	35 056 (11 392)	20.2 (20.5)	3.5 (4.2)
*P* value for *t* test	NA	.03	<.001	>.99	>.99
STI screening					
Prepandemic, mean (SD)	79 018	31.7 (11.6)	32 981 (11 051)	25.7 (24.9)	3.7 (4.8)
Post, mean (SD)	44 676	31.4 (11.2)	33 232 (11 036)	25.0 (24.4)	3.6 (4.5)
*P* value for *t* test	NA	.002	.004	<.001	.02
LARC insertion					
Prepandemic, mean (SD)	9726	31.4 (9.3)	35 170 (10 800)	20.1 (18.4)	3.6 (4.2)
Postpandemic, mean (SD)	5754	31.1 (9.0)	35 419 (10 681)	19.5 (18.2)	3.5 (4.0)
*P* value for *t* test	NA	>.99	>.99	>.99	>.99
Pharmacy-obtained contraception					
Prepandemic, mean (SD)	169 420	29.4 (8.8)	34 625 (10 841)	18.4 (18.8)	3.3 (4.1)
Postpandemic, mean (SD)	89 488	29.4 (8.9)	34 976 (11 067)	18.7 (18.9)	3.3 (4.0)
*P* value for *t* test	NA	.58	<.001	.01	>.99

Abbreviation: NA, not applicable.

^a^ For each preventive service, we report the mean and SD of the covariates (age, zip code per-capita income, zip code percentage non-White, and zip code percentage not English proficient) among women receiving each service in the prepandemic vs postpandemic period. The prepandemic months are January 2019 to February 2020, and postpandemic months are March to December 2020. We report the results of 2-sided *t* tests of differences between the mean values of each covariate in the prepandemic vs postpandemic periods for each service. All *P* values are reported using the Bonferroni correction for multiple comparisons; by convention, *P* values greater than 1 after correction are reported as >.99.

## Discussion

Utilization of women’s preventive care services among a commercially insured population of women in Michigan was significantly lower in 2020 relative to 2019. For services that require an in-person visit or procedure, these declines were temporally associated with the timing of clinical service shutdowns implemented across the state in response to the initial surge of COVID-19 cases and hospitalizations during April through June 2020.

While claim rates for these services returned to close to 2019 levels by July 2020, with the exception of breast cancer screening, they did not subsequently increase to higher rates in later months relative to 2019. For breast cancer screening, even though rates increased slightly above 2019 levels in the last few months of the year, the overall odds that a woman received breast cancer screening remained 20% lower in 2020 relative to 2019. This could reflect capacity constraints in the health care system, a shift to longer intervals between services, or overall reduced demand for preventive care among patients. Overall, these results suggest that some women may be experiencing delays in recommended preventive care.

In a post hoc analysis, we found suggestive evidence that the sociodemographic distribution of women receiving each type of service changed during the pandemic, although the absolute changes we detected were small. For 3 of the 5 services (cervical cancer screening, STI screening, and pharmacy-provided contraception), women receiving these services in the postpandemic period lived in zip codes with higher per-capita income, suggesting a possible relative decline in utilization of these services among women in lower-income zip codes. Similarly, for 2 services (breast cancer screening and STI screening), women in the postpandemic period receiving these services were more likely to live in zip codes with lower percentages of non-White residents and lower percentages of non–English-proficient speakers. Among the 15 pairwise tests we conducted evaluating the zip code characteristics of income, race, and English-speaking population, 7 suggested a shift in utilization that may reflect worsening disparities in use of these services. Only 1 comparison suggested a shift in the opposite direction. This was an exploratory analysis, and many of the statistically significant differences were of a small magnitude. However, these findings raise the concerning possibility that the pandemic worsened preexisting disparities in access to these key services among vulnerable women. Future work is needed to further explore shifts in utilization to determine whether the differences we detect here are consistent and clinically significant.

The long-term outcomes of these preventive care delays are not yet known and are likely specific to each type of service. For cancer screening, most women who canceled or deferred recommended screening tests may be able to reschedule these services relatively quickly. However, if patients whose screening tests were canceled are unable to reschedule or are lost to follow-up, the pandemic could produce clinically relevant delays in cancer detection and treatment.

Some prior utilization of cancer screening and in-person visits for contraception prescriptions may have been inappropriate or low in clinical value.^18,19,20^ If women’s health clinicians can effectively identify and prioritize higher-risk women to reschedule their screening tests, and correspondingly defer inappropriate visits or screening tests in lower-risk women, this approach could mitigate potential harms owing to care delays.

Reproductive health services, such as STI testing or access to contraception, may have different implications. Missed or delayed diagnoses of STIs have the potential to cause immediate and delayed harm to patients and their current or future sexual partners.^21,22^ Similarly, an unwanted pregnancy is an immediate poor health outcome. For this reason, the National Quality Forum has designated LARC utilization rates less than 1% to 2% as an important indicator of unmet need for family planning services, with the important caveat that absolute LARC utilization rates will vary among different populations based on patient preferences.^23^ Reductions in pharmacy-obtained contraception may be associated with social distancing recommendations reducing the frequency of sexual activity and need for contraception during the pandemic, thereby also reducing rates of risky sexual behavior that might prompt STI screening; recent survey data support this possibility.^24^ The ultimate outcome of the reduced rates of STI screening and contraceptive use that we observed on overall STI prevalence and rates of unwanted pregnancy is an important subject for future research.

### Limitations

This study has several limitations. The data we used from a commercial health plan in Michigan may not be generalizable to other states or women with Medicaid, Medicare, or ACA Marketplace health plans. The results only estimate changes in utilization among individuals enrolled in insurance and do not include changes in utilization among women who lost their health insurance during the pandemic. However, the average monthly enrollment in the BCBSM health plan only declined by 1.3% between the prepandemic and postpandemic periods, suggesting that for this population, our estimates do not significantly underestimate total changes in utilization among women. The data lack important individual-level covariates, including income, race/ethnicity, and language spoken; we have instead used zip code–level covariates as imperfect proxies. In addition, the data only describe utilization through December 2020; subsequent data may show overall increases in utilization as enrollees reschedule missed or deferred care. Lastly, the most recent billing date for the available data was January 2021, and because approximately 5% of utilization is billed 2 months or more after the date of service, the utilization rates for December 2020 may slightly underestimate total utilization for this final study month.

## Conclusions

In this cross-sectional study of women enrolled in a large US commercial health maintenance organization plan, significant declines in use of women’s preventive health services during the COVID-19 pandemic were found that were relatively short lived except for persistent declines in hormonal contraceptives obtained from pharmacies. As a result, the likelihood of women receiving preventive health services in 2020 was significantly lower than in 2019. These findings suggest that the pandemic may be associated with worsened racial and income inequality in access to preventive services among women with commercial insurance. Further research is needed to understand how these disruptions in care have affected disparities in access to care and women’s health outcomes.

## Notes

Supplement. **eAppendix 1.** Diagnostic, procedural, and drug codes used to identify health services **eAppendix 2.** Adjusted odds ratios of utilization of women’s preventive health services in July 2020 to December 2020 compared with July 2019 to December 2019
