# Supplementary material for: Utilization of Women’s Preventive Health Services During the COVID-19 Pandemic
Source: JAMA Health Forum. 2021 Jul 2;2(7):e211408. doi: 10.1001/jamahealthforum.2021.1408 (PMC8796922; doi:10.1001/jamahealthforum.2021.1408)
Supplement: Supplement. — eAppendix 1. Diagnostic, procedural, and drug codes used to identify health services eAppendix 2. Adjusted odds ratios of utilization of women’s preventive health services in July 2020 to December 2020 compared with July 2019 to December 2019 [file jamahealthforum-e211408-s001.pdf]

Supplemental Online Content

Becker NV, Moniz MH, Tipirneni R, Dalton VK, Ayanian JZ. Utilization of women's preventive health services during the COVID-19 pandemic. *JAMA Health Forum*. 2021;2(7):e211408. doi:10.1001/jamahealthforum.2021.1408

eAppendix 1. Diagnostic, procedural, and drug codes used to identify health services

eAppendix 2. Adjusted odds ratios of utilization of women's preventive health services in July 2020 to December 2020 compared with July 2019 to December 2019

This supplemental material has been provided by the authors to give readers additional information about their work.

© 2021 Becker NV et al. *JAMA Health Forum*.

eAppendix 1. Diagnostic, procedural, and drug codes used to identify health services

Preventive service	Type of Code	Code	Description
breast cancer screening	ICD-10-CM	Z1231	Encounter for screening mammogram for malignant neoplasm of breast
breast cancer screening	CPT	77067	Screening mammography, bilateral (2-view study of each breast), including computer-aided detection (CAD) when performed
breast cancer screening	CPT	77063	Screening digital breast tomosynthesis, bilateral
cervical cancer screening	ICD-10-CM	Z124	Screening for malignant neoplasms of the cervix (routine cervical pap smear)
cervical cancer screening	ICD-10-CM	Z1151	Special screening examination for human papillomavirus (HPV)
cervical cancer screening	HCPCS	Q0091	Screening papanicolaou smear; obtaining, preparing and conveyance of cervical or vaginal smear to laboratory
cervical cancer screening	HCPCS	G0123	Screening cytopathology, cervical or vaginal (any reporting system), collected in preservative fluid, automated thin layer preparation, screening by cytotechnologist under physician supervision
cervical cancer screening	HCPCS	G0124	Screening cytopathology, cervical or vaginal (any reporting system), collected in preservative fluid, automated thin layer preparation, requiring interpretation by physician
cervical cancer screening	HCPCS	G0143	Screening cytopathology, cervical or vaginal (any reporting system), collected in preservative fluid, automated thin layer preparation, with manual screening and rescreening by cytotechnologist under physician supervision
cervical cancer screening	HCPCS	G0144	Screening cytopathology, cervical or vaginal (any reporting system), collected in preservative fluid, automated thin layer preparation, with screening by automated system, under physician supervision
cervical cancer screening	HCPCS	G0145	Screening cytopathology, cervical or vaginal (any reporting system), collected in preservative fluid, automated thin layer preparation, with screening by automated system and manual rescreening under physician supervision
cervical cancer screening	HCPCS	G0147	Screening cytopathology smears, cervical or vaginal, performed by automated system under physician supervision
cervical cancer screening	HCPCS	G0141	Screening cytopathology smears, cervical or vaginal, performed by automated system, with manual rescreening, requiring interpretation by physician
cervical cancer screening	HCPCS	G0148	Screening cytopathology smears, cervical or vaginal, performed by automated system with manual rescreening
cervical cancer screening	HCPCS	P3000	Screening papanicolaou smear, cervical or vaginal, up to three smears, by technician under physician supervision
cervical cancer screening	HCPCS	P3001	Screening papanicolaou smear, cervical or vaginal, up to three smears, requiring interpretation by physician
cervical cancer screening	CPT	88141	Cytopathology, cervical or vaginal (any reporting system), requiring interpretation by physician
cervical cancer screening	CPT	88142	Cytopathology, cervical or vaginal (any reporting system), collected in preservative fluid, automated thin layer preparation; manual screening under physician supervision
cervical cancer screening	CPT	88143	Cytopathology, cervical or vaginal (any reporting system), collected in preservative fluid, automated thin layer preparation; with manual screening and rescreening under physician supervision
cervical cancer screening	CPT	88147	Cytopathology smears, cervical or vaginal; screening by automated system under physician supervision
cervical cancer screening	CPT	88148	Cytopathology smears, cervical or vaginal; screening by automated system with manual rescreening under physician supervision
cervical cancer screening	CPT	88150	Cytopathology, slides, cervical or vaginal; manual screening under physician supervision
cervical cancer screening	CPT	88152	Cytopathology, slides, cervical or vaginal; with manual screening and computer-assisted rescreening under physician supervision
cervical cancer screening	CPT	88153	Cytopathology, slides, cervical or vaginal; with manual screening and rescreening under physician supervision

© 2021 Becker NV et al. *JAMA Health Forum*.

cervical cancer screening	CPT	88154	Cytopathology, slides, cervical or vaginal; with manual screening and computer-assisted rescreening using cell selection and review under physician supervision
cervical cancer screening	CPT	88164	Cytopathology, slides, cervical or vaginal (the Bethesda System); manual screening under physician supervision
cervical cancer screening	CPT	88165	Cytopathology, slides, cervical or vaginal (the Bethesda System); with manual screening and rescreening under physician supervision
cervical cancer screening	CPT	88166	Cytopathology, slides, cervical or vaginal (the Bethesda System); with manual screening and computer-assisted rescreening under physician supervision
cervical cancer screening	CPT	88167	Cytopathology, slides, cervical or vaginal (the Bethesda System); with manual screening and computer-assisted rescreening using cell selection and review under physician supervision
cervical cancer screening	CPT	88174	Cytopathology, cervical or vaginal (any reporting system), collected in preservative fluid, automated thin layer preparation; screening by automated system, under physician supervision
cervical cancer screening	CPT	88175	Cytopathology, cervical or vaginal (any reporting system), collected in preservative fluid, automated thin layer preparation; with screening by automated system and manual rescreening or review, under physician supervision
cervical cancer screening	CPT	87623	HPV genotyping, low risk
cervical cancer screening	CPT	87624	HPV genotyping, high risk
cervical cancer screening	CPT	87625	HPV genotyping 16, 18, 45
STI screening	ICD-10-CM	Z113	Encounter for screening for infections with a predominantly sexual mode of transmission
STI screening	ICD-10-CM	Z114	Encounter for screening for human immunodeficiency virus [HIV]
STI screening	ICD-10-CM	Z7251	High risk heterosexual behavior
STI screening	ICD-10-CM	Z7252	High risk homosexual behavior
STI screening	ICD-10-CM	Z7253	High risk bisexual behavior
STI screening	ICD-10-CM	Z725	High-risk sexual behavior
STI screening	HCPCS	G0445	High intensity behavioral counseling to prevent sexually transmitted infection; face-to-face, individual, includes: education, skills training and guidance on how to change sexual behavior; performed semi-annually, 30 minutes
STI screening	CPT	86631	Antibody; Chlamydia
STI screening	CPT	86632	Antibody; Chlamydia, IgM
STI screening	CPT	87110	Culture, chlamydia, any source
STI screening	CPT	87270	Infectious agent antigen detection by immunofluorescent technique; Chlamydia trachomatis
STI screening	CPT	87320	Infectious agent antigen detection by enzyme immunoassay technique, qualitative or semiquantitative, multiple-step method; Chlamydia trachomatis
STI screening	CPT	87490	Infectious agent detection by nucleic acid (DNA or RNA); Chlamydia trachomatis, direct probe technique
STI screening	CPT	87491	Infectious agent detection by nucleic acid (DNA or RNA); Chlamydia trachomatis, amplified probe technique
STI screening	CPT	87810	Infectious agent antigen detection by immunoassay with direct optical observation; Chlamydia trachomatis
STI screening	CPT	87800	Infectious agent detection by nucleic acid (DNA or RNA), multiple organisms; direct probe(s) technique
STI screening	CPT	87590	Infectious agent detection by nucleic acid (DNA or RNA); Neisseria gonorrhea, direct probe technique
STI screening	CPT	87591	Infectious agent detection by nucleic acid (DNA or RNA); Neisseria gonorrhea, amplified probe technique
STI screening	CPT	87850	Infectious agent antigen detection by immunoassay with direct optical observation; Neisseria gonorrhea

© 2021 Becker NV et al. *JAMA Health Forum*.

STI screening	CPT	86592	Syphilis test, non-treponemal antibody; qualitative (eg, VDRL, RPR, ART)
STI screening	CPT	86593	Syphilis test, non-treponemal antibody; quantitative
STI screening	CPT	86780	Antibody; Treponema pallidum
STI screening	CPT	86701	HIV-1
STI screening	CPT	86702	HIV-2
STI screening	CPT	86703	HIV-1 and HIV-2, single result
STI screening	CPT	87389	HIV-1 antigen(s), with HIV-1 and HIV-2 antibodies, single result
STI screening	HCPCS	G0432	Infectious agent antibody detection by enzyme Immune assay (EIA) technique, qualitative or Semi-quantitative, multiple-step method, HIV-1 or HIV-2, screening
STI screening	HCPCS	G0433	Infectious agent antibody detection by enzyme-linked immunosorbent assay (ELISA) technique, antibody, HIV-1 or HIV2, screening
STI screening	HCPCS	G0435	Infectious agent antibody detection by rapid antibody test of oral mucosa transudate, HIV-1 or HIV-2, screening.
STI screening	HCPCS	G0475	HIV combination assay
IUD	ICD-10-CM	Z30430	Encounter for insertion of intrauterine contraceptive device
IUD	ICD-10-CM	Z30014	Encounter for initial prescription of intrauterine contraceptive device
IUD	ICD-10-CM	Z30433	Encounter for removal and reinsertion of intrauterine contraceptive device
IUD	ICD-10-PCS	0UH97HZ	Insertion of Contraceptive Device into Uterus, Via Natural or Artificial Opening
IUD	ICD-10-PCS	0UH98HZ	Insertion of Contraceptive Device into Uterus, Via Natural or Artificial Opening Endoscopic
IUD	ICD-10-PCS	0UHC7HZ	Insertion of Contraceptive Device into Cervix, Via Natural or Artificial Opening
IUD	ICD-10-PCS	0UHC8HZ	Insertion of Contraceptive Device into Cervix, Via Natural or Artificial Opening Endoscopic
IUD	ICD-10-PCS	0UH90HZ	Insertion of Contraceptive Device into Uterus, Open Approach
IUD	CPT	58300	Insertion of IUD
IUD	HCPCS	J7300	Intrauterine copper contraceptive
IUD	HCPCS	J7301	Levonorgestrel-releasing intrauterine contraceptive system, 13.5 mg
IUD	HCPCS	J7302	Levonorgestrel- releasing intrauterine contraceptive system, 52 mg
IUD	HCPCS	S4989	Contraceptive intrauterine device (e.g. progestacertiud), including implants and supplies
IUD	HCPCS	Q0090	Levonorgestrel-releasing intrauterine contraceptive system, (skyla), 13.5 mg
IUD	HCPCS	S4981	Insertion of levonorgestrel- releasing intrauterine system
IUD	HCPCS	J7297	Levonorgestrel-releasing intrauterine contraceptive system, 52 mg, 3 years duration
IUD	HCPCS	J7298	Levonorgestrel-releasing intrauterine contraceptive system, 52 mg, 5 year duration
IUD	NDC	50419042101	Mirena
IUD	NDC	50419042201	Skyla
IUD	NDC	51285020401	ParaGard
IUD	NDC	50419042208	Skyla
IUD	NDC	50419042271	Skyla
IUD	NDC	50419042301	Mirena
IUD	NDC	51285020402	ParaGard
IUD	NDC	52544003554	Liletta
IUD	NDC	00023585801	Liletta
IUD	NDC	50419042401	Kyleena
IUD	NDC	50419042408	Kyleena
IUD	NDC	50419042471	Kyleena
IUD	NDC	50419042308	Mirena
implant	ICD-10-CM	Z30017	Encounter for initial prescription of implantable subdermal contraceptive
implant	CPT	11981	Insertion, non- biodegradable drug delivery implant, Implanon or Nexplanon

© 2021 Becker NV et al. *JAMA Health Forum.*

implant	HCPS	J7306	Levonorgestrel (contraceptive) implant system, including implants and supplies
implant	HCPS	J7307	Etonogestrel [contraceptive] implant system, including implant and supplies
implant	CPT	11983	Removal with reinsertion, non- biodegradable drug delivery implant, Implanon or Nexplanon
implant	HCPS	J7306	Levonorgestrel (contraceptive) implant system, including implants and supplies
implant	HCPS	J7307	Etonogestrel [contraceptive] implant system, including implant and supplies
implant	NDC	00052027201	IMPLANON
implant	NDC	00052027401	NEXPLANON
implant	NDC	00052433001	NEXPLANON
implant	NDC	00052027480	NEXPLANON
pill	ICD-10-CM	Z30011	Encounter for initial prescription of contraceptive pills
pill	ICD-10-CM	Z3041	Encounter for surveillance of contraceptive pills
pill	HCPCS	S4993	Contraceptive pills for birth control
pill	NDC	00008111720	Lybrel
pill	NDC	00008111730	Lybrel
pill	NDC	00008251402	Lo/Ovral-28
pill	NDC	00008253505	Triphasil-21
pill	NDC	00008253601	Triphasil-28
pill	NDC	00008253605	Triphasil-28
pill	NDC	00052026106	Desogen
pill	NDC	00052028306	Cyclessa
pill	NDC	00062125100	Ortho Tri-Cyclen Lo
pill	NDC	00062125115	Ortho Tri-Cyclen Lo
pill	NDC	00062125120	Ortho Tri-Cyclen Lo
pill	NDC	00062133220	Ortho-Novum 1/50
pill	NDC	00062141116	Ortho Micronor
pill	NDC	00062141123	Ortho Micronor
pill	NDC	00062171400	Modicon
pill	NDC	00062171415	Modicon
pill	NDC	00062176100	Ortho-Novum 1/35
pill	NDC	00062176115	Ortho-Novum 1/35
pill	NDC	00062178100	Ortho-Novum 7/7/7
pill	NDC	00062178115	Ortho-Novum 7/7/7
pill	NDC	00062179600	Ortho-Cept
pill	NDC	00062179615	Ortho-Cept
pill	NDC	00062190120	Ortho-Cyclen
pill	NDC	00062190320	Ortho Tri-Cyclen
pill	NDC	00062190700	Ortho-Cyclen
pill	NDC	00062190715	Ortho-Cyclen
pill	NDC	00062191000	Ortho Tri-Cyclen
pill	NDC	00062191015	Ortho Tri-Cyclen
pill	NDC	00247052028	Tri-Norinyl
pill	NDC	00247069028	Ortho-Cyclen
pill	NDC	00247069128	Ortho Tri-Cyclen
pill	NDC	00247069228	Triphasil-28
pill	NDC	00247139828	Ovcon 50
pill	NDC	00247151328	Ovcon 35
pill	NDC	00247151628	Tri-Levlen
pill	NDC	00247151728	Desogen
pill	NDC	00247176404	Loestrin 21 1/20
pill	NDC	00247176421	Loestrin 21 1/20
pill	NDC	00247176521	Loestrin 21 1.5/30
pill	NDC	00247198621	Yasmin

© 2021 Becker NV et al. *JAMA Health Forum*.

pill	NDC	00247198628	Yasmin
pill	NDC	00247200828	Loestrin 21 1/20
pill	NDC	00247201004	Lo/Ovral-28
pill	NDC	00247201008	Lo/Ovral-28
pill	NDC	00247201028	Lo/Ovral-28
pill	NDC	00247201228	Ortho-Cyclen
pill	NDC	00247201328	Ortho-Novum 7/7/7
pill	NDC	00247214728	Ortho Tri-Cyclen Lo
pill	NDC	00247216928	Tri-Sprintec
pill	NDC	00247217028	Trivora-28
pill	NDC	00247223028	Mircette
pill	NDC	00247223528	Kariva
pill	NDC	00247226028	Ortho-Novum 1/35
pill	NDC	00247226828	Yasmin
pill	NDC	00378655053	Ethinyl Estradiol-Levonorgestrel
pill	NDC	00378727253	Norethindrone
pill	NDC	00378729253	Norethindrone
pill	NDC	00430042014	Lo Loestrin Fe
pill	NDC	00430048214	Femcon FE
pill	NDC	00430053014	Loestrin 24 Fe
pill	NDC	00430053550	Minastrin 24 Fe
pill	NDC	00430057014	Estrostep Fe
pill	NDC	00430057045	Estrostep Fe
pill	NDC	00430058014	Ovcon 35
pill	NDC	00430058045	Ovcon 35
pill	NDC	00430058114	Ovcon 35 Fe
pill	NDC	00430058514	Ovcon 50
pill	NDC	00430058545	Ovcon 50
pill	NDC	00555034458	Errin
pill	NDC	00555071558	Camila
pill	NDC	00555900867	Nortrel 0.5/35
pill	NDC	00555900942	Nortrel 1/35
pill	NDC	00555901058	Nortrel 1/35
pill	NDC	00555901258	Nortrel 7/7/7
pill	NDC	00555901467	Lessina
pill	NDC	00555901658	Sprintec
pill	NDC	00555901858	Tri-Sprintec
pill	NDC	00555902058	Portia
pill	NDC	00555902542	Junel 1/20
pill	NDC	00555902557	Junel 1/20
pill	NDC	00555902658	Junel Fe 1/20
pill	NDC	00555902742	Junel 1.5/30
pill	NDC	00555902757	Junel 1.5/30
pill	NDC	00555902858	Junel Fe 1.5/30
pill	NDC	00555903270	Tri-Legest Fe
pill	NDC	00555903458	Balziva
pill	NDC	00555904358	Apri
pill	NDC	00555904558	Aviane
pill	NDC	00555904758	Enpresse
pill	NDC	00555904958	Cryselle 28
pill	NDC	00555905058	Kariva
pill	NDC	00555905158	Velivet
pill	NDC	00555905167	Velivet
pill	NDC	00555906458	Kelnor
pill	NDC	00555906467	Kelnor

© 2021 Becker NV et al. *JAMA Health Forum*.

pill	NDC	00555906558	Tri-Lo-Sprintec
pill	NDC	00555906658	Aranelle
pill	NDC	00555906667	Aranelle
pill	NDC	00555912366	Jolessa
pill	NDC	00555913167	Ocella
pill	NDC	00555913179	Ocella
pill	NDC	00603359017	Gildagia
pill	NDC	00603359049	Gildagia
pill	NDC	00603752117	Cyclafem 1/35
pill	NDC	00603752149	Cyclafem 1/35
pill	NDC	00603752517	Cyclafem 7/7/7
pill	NDC	00603752549	Cyclafem 7/7/7
pill	NDC	00603754017	Emoquette
pill	NDC	00603754049	Emoquette
pill	NDC	00603760615	Gildess
pill	NDC	00603760648	Gildess
pill	NDC	00603760715	Gildess
pill	NDC	00603760748	Gildess
pill	NDC	00603760817	Gildess FE 1.5/30
pill	NDC	00603760917	Gildess FE 1/20
pill	NDC	00603762517	Myzilra
pill	NDC	00603762549	Myzilra
pill	NDC	00603763417	Orsythia
pill	NDC	00603763449	Orsythia
pill	NDC	00603764017	Previfem
pill	NDC	00603764217	Previfem
pill	NDC	00603766317	Tri-Previfem
pill	NDC	00603766517	Tri-Previfem
pill	NDC	23490765301	Desogen
pill	NDC	23490767001	Mircette
pill	NDC	23490769901	Lo/Ovral-28
pill	NDC	24090080184	Lo/Ovral-28
pill	NDC	24090096184	Ethinyl Estradiol-Norgestrel
pill	NDC	35356001468	Nortrel 1/35
pill	NDC	35356001568	Tri-Previfem
pill	NDC	35356002168	Ortho Tri-Cyclen
pill	NDC	35356025528	Yaz
pill	NDC	35356037028	Cryselle 28
pill	NDC	43386062030	My Way
pill	NDC	45802084054	Levonorgestrel
pill	NDC	50419040201	Yasmin
pill	NDC	50419040203	Yasmin
pill	NDC	50419040303	Safyral
pill	NDC	50419040503	Yaz
pill	NDC	50419040701	Beyaz
pill	NDC	50419040703	Beyaz
pill	NDC	50419041112	Levlen
pill	NDC	50419041128	Levlen
pill	NDC	50419043306	Tri-Levlen
pill	NDC	50419043312	Tri-Levlen
pill	NDC	50452025115	Ortho Tri-Cyclen Lo
pill	NDC	50458017115	Modicon
pill	NDC	50458017615	Ortho-Novum 1/35
pill	NDC	50458017815	Ortho-Novum 7/7/7
pill	NDC	50458019115	Ortho Tri-Cyclen

© 2021 Becker NV et al. *JAMA Health Forum*.

pill	NDC	50458019411	Ortho Micronor
pill	NDC	50458019416	Ortho Micronor
pill	NDC	50458019615	Ortho-Cept
pill	NDC	50458019715	Ortho-Cyclen
pill	NDC	50458025115	Ortho Tri-Cyclen Lo
pill	NDC	51285005866	Seasonale
pill	NDC	51285007997	Loestrin 21 1/20
pill	NDC	51285008070	Loestrin Fe 1/20
pill	NDC	51285008198	Loestrin Fe 1/20
pill	NDC	51285008297	Loestrin 21 1.5/30
pill	NDC	51285008370	Loestrin Fe 1.5/30
pill	NDC	51285008498	Loestrin Fe 1.5/30
pill	NDC	51285008787	Seasonique
pill	NDC	51285009158	Nordette
pill	NDC	51285009287	LoSeasonique
pill	NDC	51285011458	Mircette
pill	NDC	51285043165	Quartette
pill	NDC	51285054628	Cryselle 28
pill	NDC	51285076993	Plan B
pill	NDC	51285094288	Plan B One-Step
pill	NDC	51285094388	Plan B One-Step
pill	NDC	52544014331	Tilia Fe
pill	NDC	52544017572	Tilia Fe
pill	NDC	52544020431	Generess Fe
pill	NDC	52544021028	Zenchent Fe
pill	NDC	52544021928	Leena
pill	NDC	52544022829	Amethia Lo
pill	NDC	52544023528	Nor-QD
pill	NDC	52544023531	Nor-QD
pill	NDC	52544024531	Necon 1/50
pill	NDC	52544024728	Mononessa
pill	NDC	52544024828	TriNessa
pill	NDC	52544025428	Brevicon
pill	NDC	52544025928	Norinyl 1+35
pill	NDC	52544025988	Norinyl 1+35
pill	NDC	52544026528	Norinyl 1+50
pill	NDC	52544026531	Norinyl 1+50
pill	NDC	52544026829	Amethia
pill	NDC	52544026884	Amethia
pill	NDC	52544027428	Tri-Norinyl
pill	NDC	52544027431	Tri-Norinyl
pill	NDC	52544027536	Next Choice
pill	NDC	52544027928	Levora
pill	NDC	52544028754	Next Choice
pill	NDC	52544029128	Trivora-28
pill	NDC	52544029231	Zenchent Fe
pill	NDC	52544029241	Zenchent Fe
pill	NDC	52544029528	Amethyst
pill	NDC	52544038328	Zovia 1/35
pill	NDC	52544038428	Zovia 1/50
pill	NDC	52544047536	Next Choice
pill	NDC	52544055028	Necon 0.5/35
pill	NDC	52544055228	Necon 1/35
pill	NDC	52544055428	Necon 10/11
pill	NDC	52544062928	Nora-Be

© 2021 Becker NV et al. *JAMA Health Forum*.

pill	NDC	52544063028	Microgestin FE 1/20
pill	NDC	52544063128	Microgestin FE 1.5/30
pill	NDC	52544084728	Low-Ogestrel
pill	NDC	52544084828	Ogestrel-28
pill	NDC	52544089228	Jolivette
pill	NDC	52544093628	Necon 7/7/7
pill	NDC	52544094028	Azurette
pill	NDC	52544094928	Lutera
pill	NDC	52544095021	Microgestin 1/20
pill	NDC	52544095121	Microgestin 1.5/30
pill	NDC	52544095328	Zenchent Fe
pill	NDC	52544095428	Reclipsen
pill	NDC	52544095931	Caziant
pill	NDC	52544096691	Quasense
pill	NDC	52544096728	Sronyx
pill	NDC	52544098131	Zarah
pill	NDC	52544098231	Vestura
pill	NDC	52959045002	Next Choice
pill	NDC	54569067900	Lo/Ovral-28
pill	NDC	54569068500	Ortho-Novum 1/35
pill	NDC	54569068501	Ortho-Novum 1/35
pill	NDC	54569068900	Ortho-Novum 7/7/7
pill	NDC	54569068901	Ortho-Novum 7/7/7
pill	NDC	54569143900	Tri-Levlen
pill	NDC	54569384400	Levlen
pill	NDC	54569422200	Desogen
pill	NDC	54569422201	Desogen
pill	NDC	54569426900	Ortho Tri-Cyclen
pill	NDC	54569427301	Ortho-Cyclen
pill	NDC	54569481700	Zovia 1/35
pill	NDC	54569487800	Apri
pill	NDC	54569487801	Apri
pill	NDC	54569489000	Mircette
pill	NDC	54569498400	Ortho Micronor
pill	NDC	54569499700	Levora
pill	NDC	54569499800	Low-Ogestrel
pill	NDC	54569516100	Nor-QD
pill	NDC	54569534300	Plan B
pill	NDC	54569534900	Yasmin
pill	NDC	54569549300	Ortho Tri-Cyclen Lo
pill	NDC	54569549302	Ortho Tri-Cyclen Lo
pill	NDC	54569579600	TriNessa
pill	NDC	54569579700	Microgestin FE 1/20
pill	NDC	54569579800	Lutera
pill	NDC	54569581600	Mononessa
pill	NDC	54569582600	Kariva
pill	NDC	54569603200	Reclipsen
pill	NDC	54569612800	Ocella
pill	NDC	54569614400	Yaz
pill	NDC	54569614500	Plan B One-Step
pill	NDC	54569627200	Gianvi
pill	NDC	54569628000	Previfem
pill	NDC	54569628100	Tri-Previfem
pill	NDC	54868042800	Lo/Ovral-28
pill	NDC	54868044300	Ortho-Novum 1/35

© 2021 Becker NV et al. *JAMA Health Forum*.

pill	NDC	54868050200	Loestrin Fe 1.5/30
pill	NDC	54868050700	Nordette
pill	NDC	54868050801	Ortho-Novum 7/7/7
pill	NDC	54868050901	Ovcon 35
pill	NDC	54868051600	Tri-Norinyl
pill	NDC	54868151200	Loestrin Fe 1/20
pill	NDC	54868156400	Levlen
pill	NDC	54868231600	Seasonale
pill	NDC	54868260600	Ortho-Cyclen
pill	NDC	54868270100	Ortho-Cept
pill	NDC	54868377200	Ovcon 50
pill	NDC	54868386300	Desogen
pill	NDC	54868394800	Estrostep Fe
pill	NDC	54868409300	Ortho Tri-Cyclen
pill	NDC	54868423900	Trivora-28
pill	NDC	54868436900	Ortho Micronor
pill	NDC	54868453800	Necon 0.5/35
pill	NDC	54868459000	Yasmin
pill	NDC	54868460700	Levora
pill	NDC	54868473000	Ortho Tri-Cyclen Lo
pill	NDC	54868473100	Mircette
pill	NDC	54868474200	Kariva
pill	NDC	54868474500	Microgestin FE 1.5/30
pill	NDC	54868475400	Apri
pill	NDC	54868477600	Nortrel 1/35
pill	NDC	54868481400	Camila
pill	NDC	54868482800	Sprintec
pill	NDC	54868485100	Cryselle 28
pill	NDC	54868486000	Enpresse
pill	NDC	54868491100	Cyclessa
pill	NDC	54868502800	Tri-Sprintec
pill	NDC	54868528600	Nortrel 7/7/7
pill	NDC	54868532600	Junel Fe 1/20
pill	NDC	54868535600	Aviane
pill	NDC	54868582600	TriNessa
pill	NDC	54868582800	Yaz
pill	NDC	54868594200	Kelnor
pill	NDC	55045283902	Plan B
pill	NDC	55045348506	Lo/Ovral-28
pill	NDC	55045349701	Low-Ogestrel
pill	NDC	55045349801	Errin
pill	NDC	55045378106	Tri-Sprintec
pill	NDC	55045378206	Enpresse
pill	NDC	55045378302	Plan B
pill	NDC	55289024708	Nordette
pill	NDC	55289088704	Ogestrel-28
pill	NDC	55887005228	Microgestin FE 1.5/30
pill	NDC	55887028628	Nortrel 1/35
pill	NDC	58016474701	Junel 1/20
pill	NDC	58016482701	Jolivette
pill	NDC	66993061128	Solia
pill	NDC	66993061528	Cesia
pill	NDC	68180084313	Ethinyl Estradiol-Levonorgestrel
pill	NDC	68180084413	Kurvelo
pill	NDC	68180084613	Daysee

© 2021 Becker NV et al. *JAMA Health Forum*.

pill	NDC	68180084813	Ethinyl Estradiol-Levonorgestrel
pill	NDC	68180085413	Ethinyl Estradiol-Levonorgestrel
pill	NDC	68180087611	Norethindrone
pill	NDC	68180087613	Norethindrone
pill	NDC	68180089713	Ethinyl Estradiol-Norethindrone
pill	NDC	68180089813	Wymzya Fe
pill	NDC	68180090213	Drospirenone-Ethinyl Estradiol
pill	NDC	68462030329	Heather
pill	NDC	68462030529	Norethindrone
pill	NDC	68462030929	Ethinyl Estradiol-Norgestimate
pill	NDC	68462031629	Briellyn
pill	NDC	68462031829	Viorele
pill	NDC	68462038829	Marlissa
pill	NDC	68462039429	Alyacen 1/35
pill	NDC	68462055629	Alyacen 7/7/7
pill	NDC	68462056529	Ethinyl Estradiol-Norgestimate
pill	NDC	68462063729	LEVONORGESTREL-ETH ESTRADIOL
pill	NDC	68462064693	Ashlyna
pill	NDC	00052026108	Desogen
pill	NDC	00093313491	Camrese
pill	NDC	00093614891	Camrese Lo
pill	NDC	00378655056	LEVONORGESTREL AND ETHINYL ESTRADIOL
pill	NDC	00378727753	NORGESTIMATE AND ETHINYL ESTRADIOL
pill	NDC	00378728098	NORETHINDRONE ACETATE AND ETHINYL ESTRADIOL
pill	NDC	00378728398	NORETHINDRONE ACETATE AND ETHINYL ESTRADIOL
pill	NDC	00378728590	LEVONORG-ETH ESTRAD ETH ESTRAD
pill	NDC	00378728756	LEVONORGESTREL AND ETHINYL ESTRADIOL
pill	NDC	00378729656	DESOGESTREL AND ETHINYL ESTRADIOL AND ETHINYL
pill	NDC	00378729853	LEVONORGESTREL-ETH ESTRADIOL
pill	NDC	00378730053	DROSPIRENONE AND ETHINYL ESTRADIOL
pill	NDC	00430042060	Lo Loestrin Fe
pill	NDC	00430042095	Lo Loestrin Fe
pill	NDC	00430048295	FEMCON Fe
pill	NDC	00430053060	Loestrin 24 Fe
pill	NDC	00430053095	Loestrin 24 Fe
pill	NDC	00430057060	ESTROSTEP Fe
pill	NDC	00781407515	Drospirenone/Ethinyl Estradiol/Levomefolate Calcium and Levomefolate Calcium
pill	NDC	00781410352	Drospirenone/Ethinyl Estradiol/Levomefolate Calcium and Levomefolate Calcium
pill	NDC	00781557515	Vienva TM
pill	NDC	16714034001	LEVONEST
pill	NDC	16714034002	LEVONEST
pill	NDC	16714034003	LEVONEST
pill	NDC	16714034601	Dasetta 7/7/7
pill	NDC	16714034602	Dasetta 7/7/7
pill	NDC	16714034603	Dasetta 7/7/7
pill	NDC	16714034802	Dasetta 1/35
pill	NDC	16714034803	Dasetta 1/35
pill	NDC	16714035901	FALMINA
pill	NDC	16714035902	FALMINA
pill	NDC	16714036001	Mono-Linyah
pill	NDC	16714036002	Mono-Linyah
pill	NDC	16714036003	Mono-Linyah
pill	NDC	16714036301	Tri-Linyah

© 2021 Becker NV et al. *JAMA Health Forum*.

pill	NDC	16714036302	Tri-Linyah
pill	NDC	16714036303	Tri-Linyah
pill	NDC	16714036501	ELINEST
pill	NDC	16714036502	ELINEST
pill	NDC	16714036503	ELINEST
pill	NDC	16714036603	SETLAKIN
pill	NDC	16714037001	Wera
pill	NDC	16714037002	Wera
pill	NDC	16714037004	Wera
pill	NDC	16714040701	Larin 1.5/30
pill	NDC	16714040702	Larin 1.5/30
pill	NDC	16714040704	Larin 1.5/30
pill	NDC	16714041601	Larin 24 Fe
pill	NDC	16714041602	Larin 24 Fe
pill	NDC	16714041603	Larin 24 Fe
pill	NDC	16714041604	Larin 24 Fe
pill	NDC	16714044001	DEBLITANE
pill	NDC	16714044002	DEBLITANE
pill	NDC	16714044003	DEBLITANE
pill	NDC	16714044101	SHAROBEL
pill	NDC	16714044102	SHAROBEL
pill	NDC	16714044103	SHAROBEL
pill	NDC	16714046401	Juleber
pill	NDC	16714046402	Juleber
pill	NDC	16714046403	Juleber
pill	NDC	16714046404	Juleber
pill	NDC	21695040701	Trinessa
pill	NDC	21695068528	Tilia FE
pill	NDC	21695077001	Tri-Sprintec
pill	NDC	21695085501	Enpresse
pill	NDC	21695085701	Necon
pill	NDC	21695099528	Aviane
pill	NDC	34908062051	LEVONEST
pill	NDC	34908062053	LEVONEST
pill	NDC	34908062056	LEVONEST
pill	NDC	50090015901	Ortho-Novum
pill	NDC	50419040300	Safyral
pill	NDC	50419040370	Safyral
pill	NDC	50419040375	Safyral
pill	NDC	50419040700	Beyaz
pill	NDC	50419040770	Beyaz
pill	NDC	50419040775	Beyaz
pill	NDC	50458017820	Ortho-Novum
pill	NDC	50458019120	Ortho Tri Cyclen
pill	NDC	50458019423	Ortho Micronor
pill	NDC	50458019720	Ortho Cyclen
pill	NDC	51285012870	Loestrin FE
pill	NDC	52544005431	Tilia Fe
pill	NDC	52544008728	TriNessa Lo
pill	NDC	52544016528	Necon
pill	NDC	52544016731	Microgestin 24 Fe
pill	NDC	52544022891	Amethia Lo
pill	NDC	52544038331	Zovia 1/35E-28
pill	NDC	52544038431	Zovia 1/50E-28
pill	NDC	52544055031	Necon 0.5/35

© 2021 Becker NV et al. *JAMA Health Forum*.

pill	NDC	52544055231	NECON 1/35
pill	NDC	52544055431	Necon 10/11
pill	NDC	52544084731	Low-Ogestrel
pill	NDC	52544098228	Vestura
pill	NDC	54868404500	Necon
pill	NDC	54868424000	Zovia 1-35E
pill	NDC	54868474400	Microgestin FE
pill	NDC	54868477800	Zovia 1-50E
pill	NDC	54868485000	Low-Ogestrel
pill	NDC	54868503100	Velivet
pill	NDC	54868592200	Ocella
pill	NDC	54868593500	Junel FE
pill	NDC	54868604400	Jolessa
pill	NDC	54868610000	Loestrin 24 Fe
pill	NDC	54868616100	Femcon FE
pill	NDC	54868616200	Gianvi
pill	NDC	54868621000	Lutera
pill	NDC	54868627200	Junel
pill	NDC	54868627300	Zenchent
pill	NDC	54868627400	Tilia FE
pill	NDC	54878727500	LoSeasonique
pill	NDC	54868627600	Seasonique
pill	NDC	57297087713	Jencycla
pill	NDC	61786038206	desogestrel and ethinyl estradiol
pill	NDC	61786038506	Caziant
pill	NDC	63187005428	MonoNessa
pill	NDC	63187045828	Tri-Sprintec
pill	NDC	66116043628	Tri-Sprintec
pill	NDC	66116047028	Gianvi
pill	NDC	68180083713	TRI-LO-MARZIA
pill	NDC	68180086413	Blisovi 24 Fe
pill	NDC	68180086513	Blisovi Fe 1/20
pill	NDC	68180086613	Blisovi Fe 1.5/30
pill	NDC	68180088013	Bekyree
pill	NDC	68180090313	Kaitlib Fe
pill	NDC	68258500502	Necon 777
pill	NDC	68462013281	Norethindrone Acetate and Ethinyl Estradiol
pill	NDC	68462065629	Norethindrone Acetate and Ethinyl Estradiol
pill	NDC	68462065690	Norethindrone Acetate and Ethinyl Estradiol
pill	NDC	68462065729	Norethindrone Acetate and Ethinyl Estradiol
pill	NDC	68462065790	Norethindrone Acetate and Ethinyl Estradiol
pill	NDC	68462067295	levonorgestrel and ethinyl estradiol
pill	NDC	68462071929	norgestimate and ethinyl estradiol
pill	NDC	68462072029	drospirenone and ethinyl estradiol
pill	NDC	76388028301	CYCLESSA
pill	NDC	76388028306	CYCLESSA
pill	NDC	76413010428	Cyclafem 7/7/7
pill	NDC	76413010528	Cyclafem 1/35
pill	NDC	76413011128	Levonorgestrel and Ethinyl Estradiol
pill	NDC	76413011628	Myzilra
pill	NDC	76413011828	Norethindrone
pill	NDC	76413012128	Previfem
pill	NDC	76413012828	Tri-Sprintec
pill	NDC	76413013028	Yasmin
pill	NDC	00378728353	NORETHIN-ETH ESTRA FERROUS FUM

© 2021 Becker NV et al. *JAMA Health Forum*.

pill	NDC	00378728753	LEVONORGESTREL-ETH ESTRADIOL
pill	NDC	00378729653	DESOGESTR-ETH ESTRAD ETH ESTRA
pill	NDC	00430053750	Lo Minastrin FE
pill	NDC	16714007304	NORETHINDRONE
pill	NDC	16714035903	Falmina
pill	NDC	16714036704	DESOGESTREL-ETHINYL ESTRADIOL
pill	NDC	16714040402	Pimtrea
pill	NDC	16714040404	Pimtrea
pill	NDC	16714040501	Larin FE
pill	NDC	16714040504	Larin FE
pill	NDC	16714040601	Larin FE
pill	NDC	16714040604	Larin FE
pill	NDC	16714040803	Larin
pill	NDC	16714041304	NORETHINDRONE
pill	NDC	50419040903	Natazia
pill	NDC	65162031684	Lomedia 24 FE
pill	NDC	65162034784	Zenchent Fe
pill	NDC	68180087513	Vyfemla
pill	NDC	68180087711	Jencycla
pill	NDC	68180087713	Jencycla
pill	NDC	68180088613	Nikki
pill	NDC	68180089211	Pirmella
pill	NDC	68180089313	Pirmella
pill	NDC	75854060101	FALESSA 1 MG TABLET
pill	NDC	63187074828	NORETHINDRONE TAB 0.35 MG
pill	NDC	51862010206	CAMILA® TABLETS 6X28
pill	NDC	00093542362	GIANVI 3 MG-0.02 MG TABLET
pill	NDC	51862003603	ZARAH
pill	NDC	51862003601	DROSPIRENONE-ETHINYL ESTRADIOL TAB 3-0.03 MG
pill	NDC	51862026006	ZOVIA® 1/35 TABLETS 6X28
pill	NDC	00378730653	ETHYNODIOL DIACETATE & ETHINYL ESTRADIOL TAB 1 MG-50
pill	NDC	51862002801	LEVONORGESTREL & ETHINYL ESTRADIOL TAB 0.1 MG-20 MCG
pill	NDC	51862054506	SRONYX® TABLETS 6X28
pill	NDC	69238153106	LEVONORGESTREL & ETHINYL ESTRADIOL TAB 0.1 MG-20 MCG
pill	NDC	51862002806	LEVONORGESTREL & ETHINYL ESTRADIOL TAB 0.1 MG-20 MCG
pill	NDC	51862009706	LEVONORGESTREL & ETHINYL ESTRADIOL TAB 0.15 MG-30 MCG
pill	NDC	51862031801	NORETHINDRONE & ETHINYL ESTRADIOL TAB 0.5 MG-35 MCG
pill	NDC	51862031803	NECON® 0.5/35 TABLETS 3X28
pill	NDC	51862000706	NORETHINDRONE ACE & ETHINYL ESTRADIOL TAB 1 MG-20 MCG
pill	NDC	68462013279	NORETHINDRONE ACE & ETHINYL ESTRADIOL TAB 1 MG-20 MCG
pill	NDC	51862027906	MICROGESTIN® 1.5/30 TABLETS 6X21
pill	NDC	51862027901	NORETHINDRONE ACE & ETHINYL ESTRADIOL TAB 1.5 MG-30 MCG
pill	NDC	51862056406	LOW-OGESTREL® TABLETS 6X28
pill	NDC	51862056401	NORGESTREL & ETHINYL ESTRADIOL TAB 0.3 MG-30 MCG
pill	NDC	69238155106	NORGESTIMATE & ETHINYL ESTRADIOL TAB 0.25 MG-35 MCG
pill	NDC	52544029841	DROSPIRENONE-ETHINYL ESTRAD-LEVOMEFOLATE TAB 3-0.02-0.451 MG
pill	NDC	52544029831	DROSPIRENONE-ETHINYL ESTRAD-LEVOMEFOLATE TAB 3-0.02-0.451 MG
pill	NDC	00378729753	NORETHINDRONE & ETHINYL ESTRADIOL-FE CHEW TAB 0.4 MG-35 MCG
pill	NDC	68180087311	NORETHINDRONE & ETHINYL ESTRADIOL-FE CHEW TAB 0.4 MG-35 MCG
pill	NDC	68180087313	NORETHINDRONE & ETHINYL ESTRADIOL-FE CHEW TAB 0.4 MG-35 MCG

© 2021 Becker NV et al. *JAMA Health Forum*.

pill	NDC	00023586228	NORETHINDRONE ACE-ETHINYL ESTRADIOL-FE CAP 1 MG-20 MCG (24)
pill	NDC	00023586230	NORETHINDRONE ACE-ETHINYL ESTRADIOL-FE CAP 1 MG-20 MCG (24)
pill	NDC	51862001201	NORETHINDRONE ACE & ETHINYL ESTRADIOL-FE TAB 1 MG-20
pill	NDC	51862001206	NORETHINDRONE ACE & ETHINYL ESTRADIOL-FE TAB 1 MG-20
pill	NDC	51862029201	NORETHINDRONE ACE & ETHINYL ESTRADIOL-FE TAB 1.5 MG-30
pill	NDC	51862029206	MICROGESTIN® FE 1.5/30 TABLETS 6X28
pill	NDC	51862007206	DESOGEST-ETH ESTRAD & ETH ESTRAD TAB 0.15-0.02/0.01 MG(21/5)
pill	NDC	51862023803	CAZIANT® TABLETS 3X28
pill	NDC	51862051006	TRIVORA® TABLETS 6X28
pill	NDC	51862047006	TRI-NORINYL® TABLETS 6X28
pill	NDC	51862047106	LEENA™ TABLETS 6X28
pill	NDC	63187075428	NORGESTIMATE-ETH ESTRAD TAB 0.18-25/0.215-25/0.25-25 MG-MCG
pill	NDC	68180083811	NORGESTIMATE-ETH ESTRAD TAB 0.18-35/0.215-35/0.25-35 MG-MCG
pill	NDC	68180083813	NORGESTIMATE-ETH ESTRAD TAB 0.18-35/0.215-35/0.25-35 MG-MCG
pill	NDC	51862028403	TILIA FE TABLETS 3x28
pill	NDC	51862004591	AMETHIA LO TABLETS 2X91
pill	NDC	00378728490	LEVONORG-ETH EST TAB 0.1-0.02MG(84) & ETH EST TAB 0.01MG(7)
pill	NDC	51862004701	AMETHIA
pill	NDC	51862004791	AMETHIA TABLETS 2X91
patch	ICD-10-CM	Z30016	Encounter for initial prescription of transdermal patch hormonal contraceptive device
patch	ICD-10-CM	Z3045	Encounter for surveillance of transdermal patch hormonal contraceptive device
patch	HCPCS	J7304	Contraceptive supply, hormone containing patch, each
patch	NDC	00062192001	Ortho Evra
patch	NDC	00062192015	Ortho Evra
patch	NDC	00062192024	Ortho Evra
patch	NDC	50458019201	Ortho Evra
patch	NDC	50458019215	Ortho Evra
patch	NDC	54569541300	Ortho Evra
patch	NDC	54868467000	Ortho Evra
patch	NDC	50458019224	Ortho Evra
patch	NDC	00378334053	Xulane
ring	ICD-10-CM	Z30015	Encounter for initial prescription of vaginal ring hormonal contraceptive
ring	ICD-10-CM	Z3044	Encounter for surveillance of vaginal ring hormonal contraceptive device
ring	HCPCS	J7303	Contraceptive supply, hormone containing vaginal ring, each
ring	NDC	00052027301	NuvaRing
ring	NDC	00052027303	NuvaRing
ring	NDC	54569586500	NuvaRing
ring	NDC	54868483201	NuvaRing
ring	NDC	55887075401	NuvaRing
ring	NDC	54868483200	NuvaRing
ring	NDC	00052027385	NuvaRing
ring	NDC	76413013103	NuvaRing
ring	NDC	65162046935	EluRyng

Abbreviations: ICD-10-CM: International Classification of Diseases diagnostic code; ICD-10-PCS: International Classification of Diseases procedural code; CPT: Current Procedural Terminology procedural code; HCPCS: Healthcare Common Procedure Coding System procedural code within medical claims; NDC: National Drug Code; STI: Sexually-transmitted infection; IUD: intrauterine device.

© 2021 Becker NV et al. *JAMA Health Forum*.

**eAppendix 2.** Adjusted odds ratios of utilization of women's preventive health services in July 2020 to December 2020 compared with July 2019 to December 2019				
	Breast cancer screening	Cervical cancer screening	STI testing	LARC insertion
N	3,627,522	5,344,555	6,176,554	3,147,123
Age range	40 to 74	21 to 65	18 to 74	18 to 45
Odds ratio July - Dec 2020 relative to July to Dec 2019 (95% CI), p-value	1.081 (1.070,1.092), $p < 0.001$	0.966 (0.954,0.979), $p < 0.001$	0.94 (0.924,0.956), $p < 0.001$	0.994 (0.946,1.044), $p = 0.804$
Each model predicts the adjusted odds ratio that an enrolled woman in the given age range has a claim for a given service in July 2020 to December 2020 relative to July 2019 to December 2019. Additional covariates include calendar month fixed effects, county fixed effects, dependent status fixed effects (primary plan-holder, spouse, child, or other dependent), age group fixed effects, and three 2019 zip-code level covariates (per capita income, percent of population that is non-white, and percent of population that is not English proficient). Robust standard errors clustered at the individual level. STI: sexually transmitted infection; LARC: long-acting reversible contraception.				

© 2021 Becker NV et al. *JAMA Health Forum*.
